# Guselkumab demonstrated an independent treatment effect in reducing fatigue after adjustment for clinical response—results from two phase 3 clinical trials of 1120 patients with active psoriatic arthritis

**DOI:** 10.1186/s13075-021-02554-3

**Published:** 2021-07-14

**Authors:** Proton Rahman, Philip J. Mease, Philip S. Helliwell, Atul Deodhar, Laure Gossec, Arthur Kavanaugh, Alexa P. Kollmeier, Elizabeth C. Hsia, Bei Zhou, Xiwu Lin, May Shawi, Chetan S. Karyekar, Chenglong Han

**Affiliations:** 1 Memorial University of Newfoundland, St. Johns, Newfoundland Canada; 2 Swedish Medical Center/Providence St. Joseph Health and University of Washington School of Medicine, Seattle, WA USA; 3 University of Leeds, Leeds, UK; 4 Oregon Health & Science University, Portland, OR USA; 5 Sorbonne Université, Institut Pierre Louis d’Epidémiologie et de Santé Publique, Paris, France; 6 AP-HP Pitié-Salpêtrière Hospital, Paris, France; 7 University of California San Diego, La Jolla, CA USA; 8 Janssen Research & Development, LLC, San Diego, CA USA; 9 Janssen Research & Development, LLC, Spring House, PA 19436 USA; 10 Univerisity of Pennsylvania School of Medicine, Philadelphia, PA USA; 11 Janssen Global Services, LLC, Horsham, PA USA

**Keywords:** Interleukin-23, p19 subunit, Biologic, Fatigue, Psoriatic arthritis, Patient-reported outcome, Mediation analysis

## Abstract

**Background:**

The interleukin-23p19-subunit inhibitor guselkumab effectively treats signs and symptoms of psoriatic arthritis (PsA). We evaluated the effect of guselkumab on fatigue.

**Methods:**

Across two phase 3 trials of guselkumab (DISCOVER-1, DISCOVER-2), patients with active PsA despite standard therapy were randomized to subcutaneous injections of guselkumab 100 mg every 4 weeks (Q4W, *N* = 373); guselkumab 100 mg at week 0, week 4, and then Q8W (*N* = 375); or placebo (*N* = 372) through week 24, after which patients in the placebo group crossed over to guselkumab Q4W. Fatigue was measured as a secondary endpoint using the Functional Assessment of Chronic Illness Therapy (FACIT)-Fatigue instrument (range 0–52, higher scores indicate less fatigue). Least-squares mean changes in FACIT-Fatigue scores were compared between treatments using a mixed-effect model for repeated measures. Mediation analysis was used to adjust for indirect effects on fatigue deriving from improvement in other outcomes, including ≥20% improvement in American College of Rheumatology criteria (ACR20; prespecified), minimal disease activity (MDA; post hoc), or C-reactive protein (CRP; post hoc).

**Results:**

Baseline mean (SD) FACIT-Fatigue scores in DISCOVER-1 (*N* = 381) and DISCOVER-2 (*N* = 739), ranging from 29.1 (9.5) to 31.4 (10.1), indicated substantial levels of fatigue relative to the United States general population (43.6 [9.4]). Across studies, mean improvements, and proportions of patients with ≥4-point improvements, in FACIT-Fatigue scores at week 24 with guselkumab Q4W and Q8W (5.6–7.6 and 54–63%, respectively) were larger vs placebo (2.2–3.6 and 35–46%). Improvement in FACIT-Fatigue scores with guselkumab was sustained from week 24 to week 52, with moderate-to-large effect sizes (Cohen’s *d* = 0.52–0.81 at week 24; 0.66–0.91 at week 52). Mediation analyses demonstrated that substantial proportions of the effects of guselkumab vs placebo on fatigue were direct effect, after adjusting for achievement of ACR20 (Q4W 69–70%, Q8W 12–36% direct effect) or MDA (72–92% across dosing regimens) response or for change in serum CRP concentrations (82–88% across dosing regimens).

**Conclusions:**

In patients with active PsA, guselkumab 100 mg Q4W or Q8W led to clinically meaningful and sustained improvements in fatigue through 1 year. A substantial portion of the improvement in FACIT-Fatigue scores induced by guselkumab was independent of effects on the achievement of other select outcomes.

**Trial registration:**

Name of the registry: ClinicalTrials.gov

Trial registrations: DISCOVER-1, NCT03162796; DISCOVER-2, NCT03158285

Date of registration: DISCOVER-1, May 22, 2017; DISCOVER-2, May 18, 2017

URLs of the trial registry record:

DISCOVER-1, https://clinicaltrials.gov/ct2/show/NCT03162796?term=NCT03162796&draw=1&rank=1

DISCOVER-2, https://clinicaltrials.gov/ct2/show/NCT03158285?term=NCT03158285&draw=2&rank=1

## Background

Psoriatic arthritis (PsA) is a chronic inflammatory arthropathy with diverse manifestations. It can affect peripheral and axial joints, be accompanied by enthesitis and/or dactylitis, and associate with psoriasis [1]. Fatigue is commonly experienced by individuals with chronic inflammatory diseases such as PsA and is associated with disease activity, pain, sleep disturbances, and depression [2, 3]. Fatigue is defined as a feeling of exhaustion, with decreased capacity for physical and mental work [4]. It includes a range of experiences, from tiredness to exhaustion, that can interfere with normal daily function and reduce health-related quality of life (HRQoL). Patients with PsA consider fatigue as one of their most important symptoms [5, 6], and moderate-to-severe fatigue is reported by up to half of PsA patients [5–9]. The importance of fatigue as a treatment target is highlighted in the European League Against Rheumatism (EULAR) recommendations for the management of PsA [10], and it has been added to the core domains for PsA assessment in clinical studies by OMERACT [11].

The mechanism underlying fatigue is complex and undefined, and it can involve physiological, psychological, and social aspects [4]. Signs and symptoms of PsA are effectively treated with biologics that block specific cytokines and reduce inflammation. Improvement of fatigue has also been evaluated in biologic clinical trials, with variable measurement tools and results [12–14]. In a recent report of a population-based cohort study, substantial fatigue remained following tumor necrosis factor-inhibitor (TNFi) therapy [15]. To develop new therapies for effective treatment of fatigue in patients with PsA, information is needed on the causality of their fatigue, as well as efficacy associated with distinct mechanisms of action.

Guselkumab (Janssen Biotech, Horsham, PA, USA), a high-affinity, human monoclonal antibody specific to the interleukin (IL)-23p19-subunit, is approved to treat patients with moderate-to-severe psoriasis and active PsA [16]. In DISCOVER-1 and DISCOVER-2, the pivotal phase 3 studies evaluating guselkumab in patients with PsA, guselkumab effectively treated the diverse manifestations of PsA, including joint signs and symptoms, physical function, skin disease, enthesitis, dactylitis, and HRQoL, with maintenance of improvements through 1 year [17–19]. We now report the efficacy of guselkumab in treating the fatigue of PsA, using a validated patient-reported outcome (PRO) instrument, the Functional Assessment of Chronic Illness Therapy-Fatigue (FACIT-Fatigue) [20, 21], through 1 year of DISCOVER-1 and DISCOVER-2. Employing mediation analysis, we also evaluated the direct effect of guselkumab on fatigue, defined as the change in FACIT-Fatigue score in patients treated with guselkumab after adjustment for select clinical outcomes, including achievement of ≥20% improvement in the American College of Rheumatology (ACR20) or minimal disease activity (MDA) responses or changes in serum C-reactive protein (CRP) concentrations.

## Methods

### Patients and study designs

DISCOVER-1 [17] and DISCOVER-2 [18] were phase 3, randomized, placebo-controlled trials of guselkumab in patients with PsA, with similar study designs. Both trials enrolled patients with active PsA, diagnosed according to the Classification Criteria for Psoriatic Arthritis (CASPAR) [22], who did not respond to or were intolerant of non-biologic disease-modifying anti-rheumatic drugs (DMARDs), apremilast, and/or non-steroidal anti-inflammatory drugs. Active PsA was defined as ≥3 tender and ≥3 swollen joints and serum CRP concentrations ≥0.3 mg/dL in DISCOVER-1 and as ≥5 tender and ≥ 5 swollen joints and serum CRP ≥0.6 mg/dL in DISCOVER-2. Prior treatment with ≤2 TNFi was permitted, but limited to approximately 30% of the study population, in DISCOVER-1 [17]. All DISCOVER-2 patients were biologic-naïve [18].

In both studies, patients were randomized to receive subcutaneous injections of guselkumab 100 mg at week 0 then every 4 weeks (Q4W), guselkumab 100 mg at weeks 0 and 4 then every 8 weeks (Q8W) with placebo at alternating 4-week intervals, or placebo Q4W. Patients in the placebo group crossed over to guselkumab 100 mg Q4W at week 24. Treatment in DISCOVER-1 continued through week 48 and in DISCOVER-2 through week 100.

### Assessment of fatigue

The FACIT-Fatigue instrument is a 13-item PRO validated to measure fatigue and its impact on daily activities and function during the previous week in patients with PsA [20, 21]. Total FACIT-Fatigue scores range from 0 to 52, with higher scores indicating less fatigue. Clinically meaningful improvement in the FACIT-Fatigue score is defined as a ≥4-point increase [21]. The mean (standard deviation [SD]) FACIT-Fatigue score, derived from limited studies in the general United States (US) population, has been reported as 43.6 (9.4) [23].

### Statistical methods

Treatment failure rules were applied to analyses of FACIT-Fatigue scores through week 24 [17, 18] and post-week 24 [19] as previously described. Changes from baseline through week 24 (placebo-controlled period) in FACIT-Fatigue scores were assessed using a mixed-effect model for repeated measures (MMRM). The MMRM adjusted for baseline FACIT-Fatigue scores and randomization stratification factors (baseline use of non-biologic DMARDs [yes/no] and prior use of TNFi [yes/no] for DISCOVER-1; baseline use of non-biologic DMARDs [yes/no] and baseline CRP [>2.0/≤2.0 mg/dL] for DISCOVER-2). Resulting least-squares (LS) mean changes and surrounding 95% confidence intervals (CIs) are reported. P values were not adjusted for multiplicity of testing.

The effect size of observed changes from baseline in FACIT-Fatigue scores at weeks 24 and 52 was calculated using Cohen’s method, by dividing the change in FACIT-Fatigue score from baseline by the SD of baseline scores. Effect sizes of 0.20, 0.50, and 0.80 indicate small, moderate, and large effects, respectively [24].

The proportions of patients achieving clinically meaningful improvement (≥4-point increase) in the FACIT-Fatigue score [21] at week 24 were compared between each guselkumab group and placebo using a Cochran–Mantel–Haenszel test (with stratification as described above); 95% CIs were derived from Wald statistics. P values were not adjusted for multiplicity of testing.

Modified cumulative distribution curves [25] were generated to show the proportions of patients achieving various levels of improvement in the FACIT-Fatigue score from ≥0 to ≥20 at week 24 by treatment group.

**Fig. 1:**
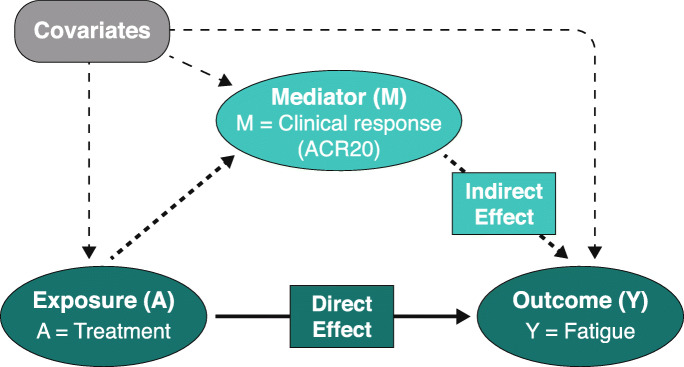
Mediation analysis. Direct effect = treatment effect on outcome independent of the effect on the mediator; indirect effect = treatment effect on outcome that is attributed to its effect on the mediator. ACR20, ≥20% improvement in American College of Rheumatology criteria

Mediation analysis [26, 27], as depicted in Fig. 1, was conducted to estimate the proportion of direct treatment effect on FACIT-Fatigue scores after adjusting for the indirect effect mediated by improvement in other selected clinical outcomes. Outcomes assessed included achievement of ACR20, as predefined, and achievement of the MDA composite endpoint [28] or changes in serum CRP concentrations, as determined post hoc. The mediation analyses employed linear regression and logistic regression models with bootstrapping to determine 95% CIs. Baseline covariates included age, sex, body mass index, baseline FACIT-Fatigue score, CRP (mg/dL), PsA duration (years), physician’s global assessment of disease (0–10-cm visual analog scale [VAS]), patient’s global assessment of arthritis (0–10-cm VAS), Health Assessment Questionnaire-Disability Index score (0–3), patient’s assessment of pain (0–10-cm VAS), swollen joint count (0–66), and tender joint count (0–68).

## Results

### Patient disposition and baseline characteristics

Of the patients enrolled in DISCOVER-1 (*N* = 381) and DISCOVER-2 (*N* = 739), 91% and 93%, respectively, completed treatment through 1 year. Patients randomized at baseline (guselkumab 100 mg Q4W, *N* = 373; guselkumab 100 mg Q8W, *N* = 375; placebo, *N* = 372) had a mean (SD) age of 46.6 (11.7), mean (SD) PsA disease duration of 5.9 (6.1) years, and substantial disease burden (Table 1). Within each study, baseline characteristics were generally consistent across randomized treatment groups. DISCOVER-2 participants had numerically higher levels of systemic inflammation and swollen and tender joint counts, per study design, and also demonstrated more skin psoriasis involvement. Mean (SD) baseline FACIT-Fatigue scores, which were consistent across randomized treatment groups and between the DISCOVER-1 and DISCOVER-2 populations, ranged from 29.1 (9.5) to 31.4 (10.1).

**Table 1:** Baseline characteristics

	DISCOVER-1			DISCOVER-2		
	Guselkumab 100 mg Q4W	Guselkumab 100 mg Q8W	Placebo	Guselkumab 100 mg Q4W	Guselkumab 100 mg Q8W	Placebo
N	128	127	126	245	248	246
Age (years)	47 (12)	49 (12)	49 (11)	46 (12)	45 (12)	46 (12)
Male, n (%)	66 (52%)	68 (54%)	61 (48%)	142 (58%)	129 (52%)	117 (48%)
BMI (kg/m^2^)	29.9 (5.5)	29.9 (6.4)	29.6 (5.7)	29.1 (5.9)	28.7 (6.3)	29.0 (6.4)
PsA disease duration (years)	6.6 (6.3)	6.4 (5.9)	7.2 (7.6)	5.5 (5.9)	5.1 (5.5)	5.8 (5.6)
Components of ACR20 and MDA						
Number of swollen joints (0–66)	8.6 (5.8)	10.9 (9.3)	10.1 (7.1)	12.9 (7.8)	11.7 (6.8)	12.3 (6.9)
Number of tender joints (0–68)	17.7 (13.1)	20.2 (14.5)	19.8 (14.4)	22.4 (13.5)	19.8 (11.9)	21.6 (13.1)
CRP (mg/dL), median (IQR)	0.6 (0.3–1.3)	0.7 (0.4–1.9)	0.8 (0.3–1.5)	1.2 (0.6–2.3)	1.3 (0.7–2.5)	1.2 (0.5–2.6)
PASI score (0–72)	9.5 (10.1)	8.4 (9.8)	7.7 (8.8)	10.8 (11.7)	9.7 (11.7)	9.3 (9.8)
HAQ-DI (0–3)	1.1 (0.6)	1.2 (0.6)	1.1 (0.6)	1.2 (0.6)	1.3 (0.6)	1.3 (0.6)
Patient’s global assessment of arthritis (VAS 0–10)	6.1 (2.0)	6.5 (2.0)	6.1 (2.2)	6.4 (1.9)	6.5 (1.9)	6.5 (1.8)
Patient’s global assessment of pain (VAS 0–10)	5.9 (2.0)	6.0 (2.1)	5.8 (2.2)	6.2 (2.0)	6.3 (2.0)	6.3 (1.8)
Physician’s global assessment (VAS 0–10)	6.2 (1.6)	6.2 (1.7)	6.3 (1.7)	6.6 (1.5)	6.6 (1.6)	6.7 (1.5)
Patients with enthesitis, n (%)	73 (57%)	72 (57%)	77 (61%)	170 (69%)	158 (64%)	178 (72%)
Leeds Enthesitis Index score (1–6)	3.0 (1.5)	2.7 (1.6)	2.8 (1.6)	3.0 (1.7)	2.6 (1.5)	2.8 (1.6)
SF-36 PCS score (0–100)	35.9 (8.3)	34.1 (7.6)	33.8 (8.5)	33.3 (7.1)	32.6 (7.9)	32.4 (7.0)
MCS score (0–100)	46.5 (9.8)	47.0 (11.1)	48.7 (9.6)	48.4 (11.0)	47.4 (10.8)	47.2 (12.0)
FACIT-Fatigue score (0–52)	31.4 (10.1)	29.5 (11.3)	30.2 (9.9)	30.8 (9.6)	29.3 (9.9)	29.1 (9.5)

Data are mean (SD) unless otherwise indicated

*ACR20* ≥20% improvement in American College of Rheumatology criteria, *BMI* body mass index, *CRP* C-reactive protein, *FACIT* Functional Assessment of Chronic Illness Therapy, *HAQ-DI* Health Assessment Questionnaire-Disability Index, *IQR* interquartile range, *MDA* minimal disease activity, *PASI* Psoriasis Area and Severity Index, *PsA* psoriatic arthritis, *SD* standard deviation, *Q4W* every 4 weeks, *Q8W* every 8 weeks, *SF-36 PCS/MCS* 36-item Short-Form physical/mental component summary, *VAS* visual analog scale

### Changes in FACIT-Fatigue scores over time through week 52

**Fig. 2:**
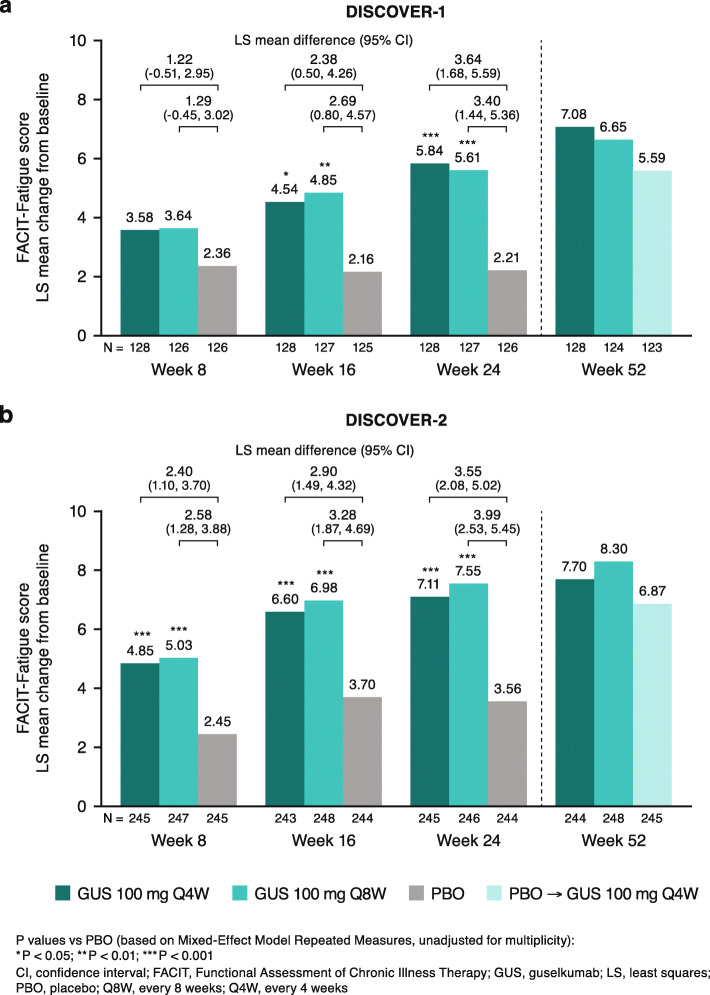
Changes from baseline in FACIT-Fatigue score through week 52 in **a** DISCOVER-1 and **b** DISCOVER-2

At week 24, mean increases (improvements) in FACIT-Fatigue scores were 5.8 and 5.6 in the guselkumab Q4W and Q8W groups, respectively, compared with 2.2 in the placebo group in DISCOVER-1; respective changes in DISCOVER-2 were 7.1 and 7.6 vs 3.6 (all *P*< 0.001, Fig. 2). Improvements were seen as early as week 8 in the larger DISCOVER-2 study of patients with more active disease at baseline, and by week 16 in the smaller DISCOVER-1 study that included TNFi-experienced patients. These substantial improvements in fatigue were maintained by guselkumab Q4W and Q8W through week 52, at which time LS mean changes were 6.7–6.9 and 7.2–8.4 across guselkumab arms in DISCOVER-1 and DISCOVER-2, respectively (Fig. 2).

The effect sizes of guselkumab Q4W and Q8W on changes in FACIT-Fatigue scores at week 24 were moderate in DISCOVER-1 (Cohen’s *d* = 0.52–0.55) and large in DISCOVER-2 (0.75–0.81); robust effect sizes were also seen at week 52 in the guselkumab Q4W (0.68–0.84) and Q8W (0.66–0.91) groups (Table 2).

**Table 2:** Guselkumab treatment effect on FACIT-Fatigue score at week 24 and week 52

	DISCOVER-1						DISCOVER-2					
	Guselkumab 100 mg Q4W		Guselkumab 100 mg Q8W		Placebo to Guselkumab 100 mg Q4W at W24		Guselkumab 100 mg Q4W		Guselkumab 100 mg Q8W		Placebo to Guselkumab 100 mg Q4W at W24	
	W 24	W 52	W 24	W 52	W 24	W 52	W 24	W 52	W 24	W 52	W 24	W 52
N	125	124	123	114	114	104	234	229	238	234	237	230
Observed mean change in FACIT-Fatigue score	5.6	6.9	5.9	7.5	2.6	6.6	7.0	7.7	8.0	8.9	3.8	7.5
(SD)	(7.8)	(8.4)	(10.4)	(9.6)	(8.3)	(9.4)	(8.6)	(9.1)	(9.9)	(9.5)	(9.0)	(9.4)
Cohen’s *d* effect size^a^	0.55	0.68	0.52	0.66	0.26	0.65	0.75	0.84	0.81	0.91	0.41	0.80

*FACIT* Functional Assessment of Chronic Illness Therapy, *Q4W* every 4 weeks, *Q8W* every 8 weeks, *SD* standard deviation, *W* week

^a^The effect size of changes from baseline in FACIT-Fatigue scores, based on the observed data, was calculated by dividing the change in FACIT-Fatigue score from baseline by the SD of baseline scores [24]

**Fig. 3:**
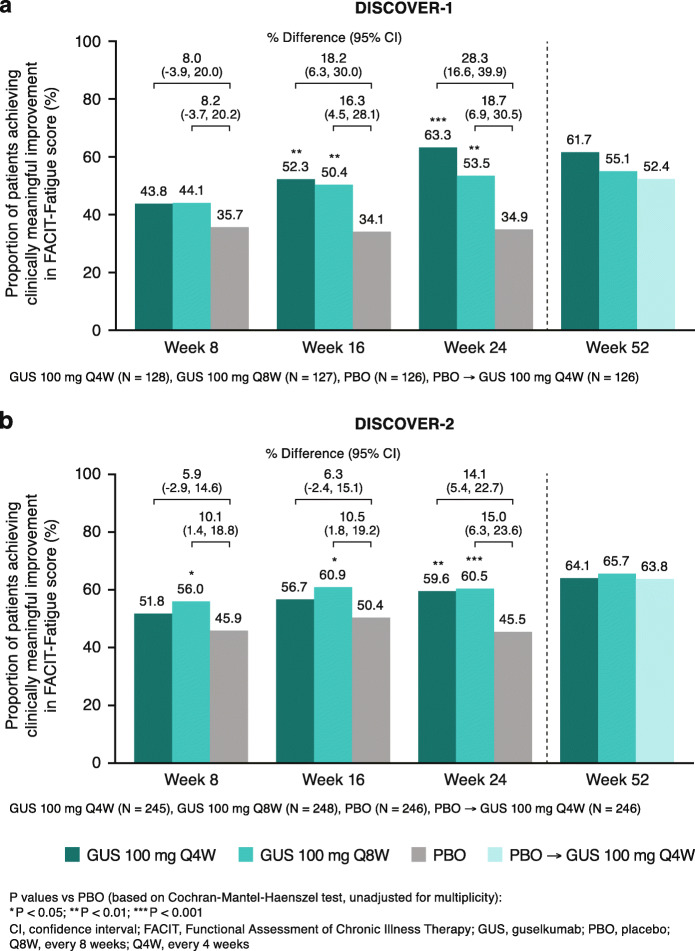
Proportion of patients achieving clinically meaningful improvement in FACIT-Fatigue score (≥4-point increase [21]) through week 52 in **a** DISCOVER-1 and **b** DISCOVER-2

Higher proportions of guselkumab- than placebo-treated patients achieved clinically meaningful improvements (≥4-point increase) in FACIT-Fatigue scores, beginning at week 8 (DISCOVER-2) or week 16 (DISCOVER-1). At week 24, significantly greater proportions of guselkumab-randomized patients achieved such improvements in FACIT-Fatigue scores when compared with placebo, i.e., and 63% (Q4W, P< 0.001) and 54% (Q8W, P<0.01) vs 35% in DISCOVER-1 and 60% (Q4W, P< 0.01) and 61% (Q8W, P< 0.001) vs 46% in DISCOVER-2. At week 52, 55–62% of patients randomized to guselkumab in DISCOVER-1 and 64–66% in DISCOVER-2 demonstrated a clinically meaningful improvement in fatigue (Fig. 3).

Patients who switched from placebo to guselkumab 100 mg Q4W at week 24 demonstrated changes from baseline and achievement of clinically meaningful improvements in FACIT-Fatigue score at week 52 comparable to those seen in patients originally randomized to guselkumab (Figs. 2 and 3).

### Cumulative distribution of improvements in FACIT-Fatigue scores at week 24

**Fig. 4:**
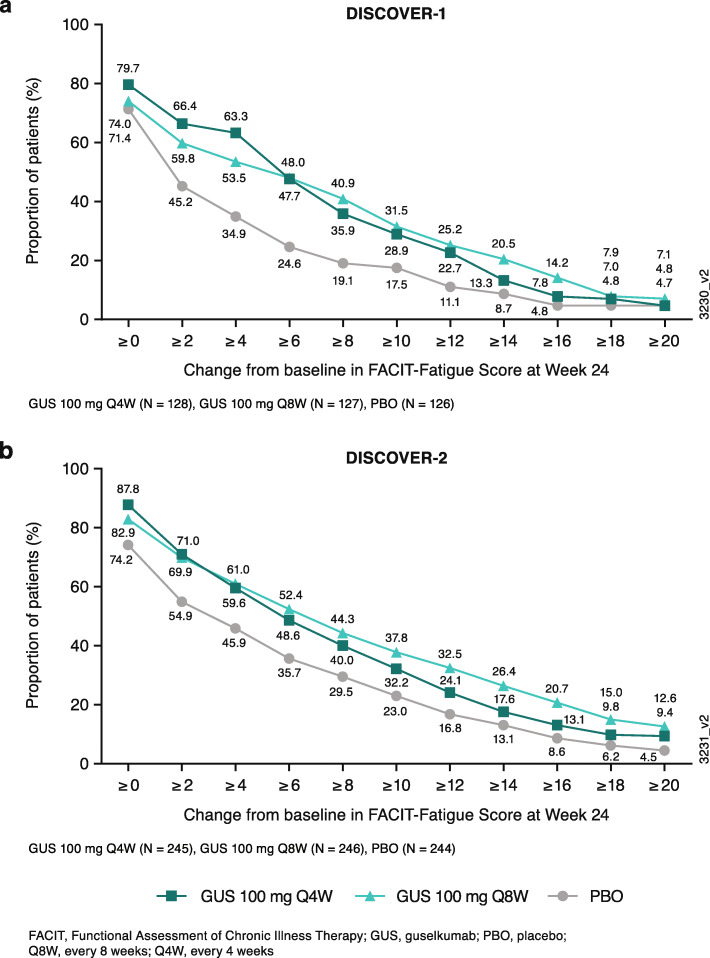
Modified cumulative distribution curves of changes in FACIT-Fatigue score at week 24 in **a** DISCOVER-1 and **b** DISCOVER-2

As shown in Fig. 4, clear separation between both guselkumab dosing regimens and placebo was observed over a range of changes from baseline in FACIT-Fatigue scores. More specifically, numerically larger proportions of patients in both the guselkumab Q4W and Q8W vs placebo groups achieved ≥2 to ≥12-point increases from baseline in FACIT-Fatigue scores at week 24.

### Mediation analyses

Results of mediation analyses indicated that improvements in the FACIT-Fatigue score observed among guselkumab-treated patients at week 24 were partially mediated indirectly through achievement of ACR20 response. With guselkumab Q4W, 69% and 70% of guselkumab’s impact on FACIT-Fatigue scores was independent of effects mediated through ACR20. For guselkumab Q8W, 12% and 36% of the effect on FACIT-Fatigue scores in DISCOVER-1 and DISCOVER-2, respectively, was independent of those mediated by achievement of ACR20 response. Independent treatment effects of guselkumab on FACIT-Fatigue scores were also observed after adjusting for other clinical outcomes, including achievement of MDA target and change from baseline in CRP concentration at week 24 (Table 3). The relative magnitudes of the direct vs indirect (mediated) effect of guselkumab on fatigue and the extent of differences between dosing regimens varied among the clinical endpoints examined.

**Table 3:** Mediation analysis of the effects of clinical responses on changes in FACIT-Fatigue scores through week 24

Clinical response		Effect	Guselkumab 100 mg Q4W vs placebo Estimate (95% CI)	Guselkumab 100 mg Q8W vs placebo Estimate (95% CI)
ACR20	DISCOVER-1	NDE	2.60 (0.58, 4.46)*	0.36 (−1.72, 2.40)
		NIE	1.20 (0.27, 2.31)*	2.75 (1.38, 4.32)*
		Total effect	3.79 (1.94, 5.44)*	3.12 (1.05, 5.15)*
		% Indirect (mediated) effect	31.5%	88.3%
		% Direct effect	68.5%	11.7%
	DISCOVER-2	NDE	2.49 (0.96, 4.14)*	1.44 (−0.11, 2.97)
		NIE	1.09 (0.42, 1.95)*	2.53 (1.62, 3.64)*
		Total effect	3.58 (2.10, 5.05)*	3.97 (2.41, 5.53)*
		% Indirect (mediated) effect	30.3%	63.7%
		% Direct effect	69.7%	36.3%
MDA	DISCOVER-1	NDE	2.84 (0.97, 4.71)*	2.34 (0.24, 4.45)*
		NIE	0.78 (0.16, 1.40)*	0.74 (0.09, 1.39)*
		Total effect	3.62 (1.76, 5.49)*	3.08 (0.99, 5.18)*
		% Indirect (mediated) effect	21.6%	24.0%
		% Direct effect	78.4%	76.0%
	DISCOVER-2	NDE	3.13 (1.63, 4.63)*	2.67 (1.09, 4.26)*
		NIE	0.28 (0.07, 0.50)*	1.06 (0.49, 1.64)*
		Total effect	3.41 (1.96, 4.86)*	3.74 (2.13, 5.34)*
		% Indirect (mediated) effect	8.3%	28.5%
		% Direct effect	91.7%	71.5%
Change in CRP	DISCOVER-1	NDE	3.19 (1.37, 5.02)*	2.51 (0.52, 4.50)*
		NIE	0.42 (−0.03, 0.86)	0.56 (−0.10, 1.22)
		Total effect	3.61 (1.74, 5.47)*	3.06 (0.95, 5.17)*
		% Indirect (mediated) effect	11.6%	18.2%
		% Direct effect	88.4%	81.8%
	DISCOVER-2	NDE	2.88 (1.36, 4.41)*	3.20 (1.62, 4.79)*
		NIE	0.49 (−0.07, 1.05)	0.60 (0.05, 1.14)*
		Total effect	3.37 (1.93, 4.81)*	3.80 (2.24, 5.36)*
		% Indirect (mediated) effect	14.5%	15.7%
		% Direct effect	85.5%	84.3%

ACR20 and MDA were dichotomous mediators; change in CRP was a continuous mediator

*ACR20* ≥20% improvement in American College of Rheumatology criteria, *CRP* C-reactive protein, *FACIT* Functional Assessment of Chronic Illness Therapy, *MDA* minimal disease activity, *NDE* natural direct effect (effect on FACIT-F beyond the effect on the clinical response), *NIE* natural indirect effect (effect on FACIT-F mediated by clinical response), *Q4W* every 4 weeks, *Q8W* every 8 weeks

*P vs placebo < 0.05

## Discussion

Guselkumab provided clinically meaningful improvements in fatigue among patients with active PsA who participated in the pivotal, phase 3, DISCOVER-1 and DISCOVER-2 trials. Guselkumab treatment effects on changes from baseline in FACIT-Fatigue scores were observed as early as week 8 in DISCOVER-2 patients, who were biologic-naïve and demonstrated more active disease at baseline, and by week 16 in DISCOVER-1 patients, one-third of whom were TNFi-experienced. The greater average improvements, and proportions of patients experiencing clinically meaningful improvement (≥4 points), in fatigue seen at week 24 were maintained in the groups of patients who continued to receive guselkumab through week 52. Guselkumab was estimated to have a moderate- (DISCOVER-1) to-large (DISCOVER-2) effect on improving FACIT-Fatigue scores through 1 year. Additionally, mediation analyses demonstrated that substantial proportions of the effects of guselkumab Q4W and Q8W vs placebo on fatigue were direct effects, after adjusting for achievement of ACR20 and MDA responses or change in serum CRP concentrations.

Fatigue is common in PsA, and moderate-to-severe fatigue has been reported by 30–50% of patients with PsA [5–9]. Although this important symptom can be neglected by physicians when assessing disease severity in rheumatological disorders [29, 30], PsA patients consider fatigue as the second or third most important health domain impacting their life and as a top priority for improvement [5, 6, 29]. Higher levels of fatigue in patients with PsA have been associated with greater work impairment, more work time missed, greater impairment while performing daily activities, higher pain scores, and diminished persistence of TNFi treatment [15, 31]. Recently, a multinational real-world PsA study demonstrated that, despite TNFi treatment, substantial fatigue persisted and was significantly associated with reduced work productivity [32]. Such findings highlight the importance of managing fatigue in PsA patients, as well as the need to assess fatigue accurately and identify effective treatment options [13].

Utilizing data derived from the 1120 PsA patients evaluated in DISCOVER-1 and DISCOVER-2, we assessed patients’ fatigue via the FACIT-Fatigue scale, an instrument that has been validated in patients with PsA [20, 21]. At baseline, patients in both DISCOVER trials had mean (SD) FACIT-Fatigue scores ranging from 29.1 (9.5) to 31.4 (10.1), compared with 43.6 (9.4), based on limited studies in a US population, and 23.9 (12.6) in patients with cancer-related anemia [23]. These data highlight the substantial levels of fatigue experienced by these PsA patients.

Numerous scales have been used to measure fatigue [20, 33–37]. The FACIT-Fatigue scale [20] and the Fatigue Numeric Rating Scale [37] have the advantage of being validated for PsA. Several controlled studies of biologics for PsA have evaluated fatigue using these validated scales, as well as more general scales [12, 38–43]. However, the description of results on fatigue from these studies is typically limited in the published literature. In a recent analysis of 880 patients with PsA and fatigue in the DANBIO registry, less than half of the patients achieved 50% improvement in their Fatigue VAS after 6 months of treatment with their initial TNFi [15]. Similarly, in a meta-analysis of patients with active rheumatoid arthritis, treatment with biologics was found to lead to only small-to-moderate improvements in fatigue [44]. Here, in addition to employing a fatigue scale validated for PsA [20, 21], and a cut-point in that scale that has been shown to define minimal clinically meaningful improvement [21], we employed modified cumulative distribution plots to demonstrate achievement of varying levels of improvement in FACIT-Fatigue scores. Importantly, in addition to a greater proportion of patients in the guselkumab vs placebo groups achieving the ≥4-point threshold of clinically meaningful improvement in FACIT-Fatigue score [21] at week 24, cumulative distribution curves also demonstrated a clear separation of both guselkumab groups from placebo over a broad range of cut-points of improvement (≥2 to ≥12) during this timeframe. In addition, by conducting analyses separately for the similarly designed DISCOVER-1 and DISCOVER-2 studies, we were able to evaluate the consistency of guselkumab’s effect on fatigue across a broad population of patients with differing degrees of active PsA at the study outset. Unfortunately, cross-study comparisons with other biologics are constrained by differences in study designs, patient populations, and scales used to measure fatigue.

Mediation analysis is a statistical technique employed to explore mechanisms underlying an observed relationship between an exposure variable and an outcome variable and how they relate to a third intermediate variable, the mediator [26, 27]. To investigate the mechanism by which guselkumab improves fatigue in PsA, we employed this computational model to distinguish direct effects of guselkumab treatment on fatigue from indirect effects, i.e., those influenced by improvement in an intermediate factor such as signs and symptoms of arthritis and/or systemic inflammation. Achievement of an ACR20 response was the prespecified mediator in these analyses, and post hoc analyses utilized achievement of MDA or changes in serum CRP levels as alternative mediators of guselkumab’s effect on FACIT-Fatigue scores. Results of the prespecified analysis indicated approximately one-third and two-thirds of the improvement in fatigue with guselkumab Q8W and Q4W, respectively, at week 24 was independent of the drug’s effect on achievement of ACR20 response and therefore not simply mediated by improvement in this particular constellation of PsA signs and symptoms. Results of the post hoc analyses assessing achievement of MDA or changes in serum CRP levels as mediators also indicated that guselkumab exerted an effect on fatigue independent from achievement of MDA response and altered systemic inflammation. Relative to mean change data, results across the conducted mediation analyses indicated the direct treatment effect of guselkumab on FACIT-Fatigue score was numerically greater with Q4W than Q8W dosing. These differences, which are inconsistent across outcome variables tested, could derive from small numerical differences in baseline disease characteristics across treatment groups and/or data variability across the various ACR and MDA components. Although findings are applied to both guselkumab dosing regimens, across the three potential mediators assessed, results are based on a statistical technique and should be interpreted with caution. Additional analyses are warranted to explore any true differences between the two guselkumab dosing regimens.

Fatigue is highly subjective, and the source is complex, undefined, and likely multidimensional [4]. Despite this, to develop more effective treatments, it is important to understand the mechanism of fatigue in PsA. Fatigue is a frequent symptom in inflammatory rheumatic diseases [2, 3] and has been postulated to be mediated in part by Th1 and Th17 pro-inflammatory cytokines in multiple sclerosis, another inflammatory autoimmune disorder [45]. Given that the IL-23/Th17 pathway is crucial to PsA pathogenesis and targeted biologic therapy [46] and that guselkumab specifically targets IL-23 through its p19 subunit, the direct effects of guselkumab on FACIT-Fatigue scores in patients with PsA could derive from its action on yet-to-be identified fatigue-specific inflammatory pathways, either alone or in combination with other factors. Further exploration is clearly needed.

Improvements in FACIT-Fatigue score in patients with PsA have also been observed with other biologics targeting different pathways [12, 39]. However, due to inherent differences in study populations, designs, and assessment tools, direct comparisons between treatments cannot be made without a head-to-head comparitive trial. As such, results of the current analysis are specific to the effects of guselkumab and cannot be compared with or extrapolated to effects of other biologic therapies. Additional research utilizing data from active-comparator studies may help to differentiate biologics in their ability to treat fatigue; such information would be useful to both physicians and patients in selecting treatments. As mentioned above, we employed computational methods in an effort to distinguish direct from indirect effects of guselkumab on fatigue. Results of these mediation analyses indicate guselkumab’s effect on fatigue is not solely dependent on its effects on achievement of ACR or MDA response, or CRP. However, these findings should be interpreted with caution given the multifactorial causation of fatigue. Future research will need to evaluate the effect of guselkumab on additional potential drivers of fatigue, e.g., activity, sleep, mental health, and anemia. Also, given the contribution of fatigue to disease burden in patients with psoriasis [47], future analyses should investigate direct treatment effects on fatigue in cohorts of patients with psoriasis. Finally, despite employing a conservative estimate of minimal clinically meaningful improvement in FACIT-Fatigue sores, we observed a relatively high placebo response rate, particularly in DISCOVER-2. However, the cumulative distribution curves confirmed that both guselkumab dosing regimens demonstrated clear separation from placebo across a range of change cut-points (i.e., ≥2 to ≥12).

## Conclusions

Taken together, results of these analyses indicate a strong impact of guselkumab on the fatigue of PsA when assessed via the FACIT-Fatigue instrument. Although further research is needed to more fully characterize the mechanism by which guselkumab improves patient fatigue, our findings may further inform treatment decisions [16], additional research on this topic, and future consensus deliberations surrounding PsA core set assessments.

## Data Availability

The data sharing policy of Janssen Pharmaceutical Companies of Johnson & Johnson is available at https://www.janssen.com/clinical-trials/transparency. As noted on this site, requests for access to the study data can be submitted through Yale Open Data Access (YODA) Project site at http://yoda.yale.edu.

## Abbreviations

ACR20: ≥ 20% improvement in American College of Rheumatology criteria
CASPAR: Classification Criteria for Psoriatic Arthritis
CI: Confidence interval
CRP: C-reactive protein
DMARD: Disease-modifying anti-rheumatic drug
EULAR: European League Against Rheumatism
FACIT: Functional Assessment of Chronic Illness Therapy
GUS: Guselkumab
HAQ-DI: Health Assessment Questionnaire-Disability Index
HRQoL: Health-related quality of life
IL: Interleukin
LS: Least-squares
MDA: Minimal disease activity
MMRM: Mixed-effect model for repeated measures
PBO: Placebo
PRO: Patient-reported outcome
PsA: Psoriatic arthritis
Q4W: Every 4 weeks
Q8W: Every 8 weeks
SD: Standard deviation
TNFi: Tumor necrosis factor-inhibitor
VAS: Visual analog scale
